# Contraindicated use of modern contraceptives among mothers from a Pelotas Birth Cohort

**DOI:** 10.11606/s1518-8787.2024058005585

**Published:** 2024-02-09

**Authors:** Gbènankpon Mathias Houvèssou, Simone Farías-Antúnez, Andréa D. Bertoldi, Mariângela Freitas da Silveira

**Affiliations:** I Universidade Federal de Pelotas Faculdade de Medicina Programa de Pós-graduação em Epidemiologia Pelotas RS Brasil Universidade Federal de Pelotas. Faculdade de Medicina. Programa de Pós-graduação em Epidemiologia. Pelotas, RS, Brasil; II Universidade Federal de Santa Catarina Faculdade de Medicina Departamento de Ciências da Saúde Florianópolis SC Brasil Universidade Federal de Santa Catarina. Faculdade de Medicina. Departamento de Ciências da Saúde. Florianópolis, SC, Brasil

**Keywords:** Contraceptive Agents, Contraindications, Progesterone, Contraceptives, Oral, Combined, Intrauterine Devices

## Abstract

**OBJECTIVE:**

To describe the prevalence of contraindicated use of combined hormonal contraceptives, progesterone-only contraceptives, and intrauterine devices in mothers participating in the 2015 Pelotas Birth Cohort according to the WHO medical eligibility criteria.

**METHODS:**

The biological mothers of children belonging to the 2015 Pelotas birth cohort who attended the 48-month follow-up were studied. The 48-month follow-up data were collected from January 1, 2019, to December 31, 2019. Contraindicated use of modern contraceptives was considered to occur when these women presented at least one of the contraindications for the use of modern contraceptives and were using these methods. The prevalence of contraindicated use was calculated according to each independent variable and their respective 95% confidence intervals (95%CI).

**RESULTS:**

The analyzed sample consisted of 3,053 women who used any modern contraceptive method. The prevalence of contraindicated use of modern contraceptives totaled 25.9% (95%CI: 24.4–27.5). Combined hormonal contraceptives showed the highest prevalence of contraindicated use (52.1%; 95%CI: 49.3–54.8). The prevalence of contraindicated use of modern contraceptives methods was greater in women with family income between one and three minimum wages, a 25–30 kg/m^2^ body mass index, indication by a gynecologist for the used method, and purchasing the contraceptive method at a pharmacy. The higher the women’s education, the lower the prevalence of inappropriate use of modern contraceptives.

**CONCLUSION:**

In total, one in four women used modern contraceptives despite showing at least one contraindication. Policies regarding women’s reproductive health should be strengthened.

## INTRODUCTION

Modern contraceptives are defined as all hormonal or barrier contraceptives, copper intrauterine devices (IUD), and female and male sterilization^1^. In more detail, they are classified into intrauterine devices and systems, subdermal implants, contraceptive patches, oral contraceptives, condoms, injectable contraceptives, emergency contraceptive pills, diaphragms and cervical caps, spermicidal agents, vaginal rings, sponges, and sterilization^2^. The use of modern contraceptive methods remains an important component in reducing fertility and maternal and child morbidity and mortality^3,4^, providing women with safe and effective control over their fertility^5^.

Although some modern contraceptives have several advantages, such as decreased menstrual blood loss, reduction of the risk of anemia through iron deficiency, and reduction of the risk of endometrial and ovarian cancer^5^, they may be inadequate for some women. Awareness in the general public about the health risks associated with their use has increased recently^5^. Women with health conditions should choose modern contraceptives that avoid worsening their health and receive health professional counseling regarding the contraindications of each alternative^6^.

The World Health Organization^7^ (WHO) Medical Eligibility Criteria for Contraceptive Use offers guidance on the safety of various contraceptive methods in relation to specific health conditions and characteristics. It provides evidence-based guidelines for prescribing contraception to women with medical contraindications and clinical illnesses. Despite these WHO eligibility criteria, a variable number of women with co-morbidities that would contraindicate the use of some contraceptives still use them. Studies carried out among women of childbearing age who use combined hormonal contraceptives (CHC) have found that the prevalence of contraindications for its use ranges from 3^8^ to 31%^9^. CHC are contraceptive methods that contain estrogen, including birth control pills, transdermal patches, and vaginal rings.

In the literature on this subject, studies have been limited to evaluating contraindications for CHC^8^. There is a paucity of studies that included progesterone-only contraceptives (POC) and IUD, which would make it possible to outline their contraindicated use. Furthermore, most studies on CHC were carried out in high-income countries. A study from Brazil and published in 2017, using data on risk surveillance and protection factors for chronic diseases by a telephone survey (VIGITEL) covering a sample of state capitals in 2008, only evaluated the use of oral contraceptives and the following contraindications for their use: hypertension, heart attack, stroke, diabetes mellitus, being a smoker and being 35 years of age or older^11^.

Family planning services in Brazil correspond to one of the seven priority areas of intervention in primary care, as defined in its Assistance Operational Standard^14^. The Ministry of Health, based on the provision of the family planning law (Law no. 9,263/96), determines, as a competence of health professionals, to assist in conception and contraception, making an effort to inform individuals about their options for both purposes and highlighting the offer of contraceptive methods that are authorized and available in Brazil^15^. Despite the mentioned conditions, negligence occurs in family planning care services in Brazil, as a consequence of a lack of following a formal protocol established. Moreover, there is a lack of appropriate services, with nurse- and doctor-centered treatment, and a lack of partnership with other reproductive health services or community groups^16^. Thus, this study was to describe the prevalence of contraindicated use of combined hormonal contraceptives, progesterone-only contraceptives, and intrauterine devices according to the WHO medical eligibility criteria in mothers participating in the 2015 Pelotas Birth Cohort. In addition to the prevalence of contraindicated use of three types of modern contraceptives (CHC, POC, and IUD) that previous studies failed to simultaneously assess, this study advances research by evaluating a considerable number of contraindications to modern contraceptives.

## METHODS

This study was conducted with mothers participating in the 2015 Pelotas birth cohort. Pelotas is located in southern Rio Grande do Sul state, Brazil, and according to an estimate from the *Instituto Brasileiro de Geografia e Estatística* (Brazilian Institute for Geography and Statistics), its population in 2020 totaled 343,132 inhabitants^17^.

The 2015 Pelotas Birth Cohort is a longitudinal study that aimed to evaluate the determinants of newborns’ health and changes to these factors over these individuals’ lifetimes. Mothers living in the urban area of Pelotas who were due to deliver in 2015 were interviewed during pregnancy. After the birth of the children, follow-ups were carried out at three, 12, 24, 48 months, and at 6–7 years. In the perinatal period, the mothers of 4,275 of the 4,333 eligible children were interviewed, and they answered questionnaires. Mothers not included were those who refused to be part of the cohort. A detailed description of the 2015 Pelotas Birth Cohort methodology is based on Hallal et al.^18^ The data generated by these questionnaires were electronically stored in the REDCap system (Research Electronic Data Capture)^19^.

Only the biological mothers of children belonging to the 2015 Pelotas Birth Cohort who answered the 48-month follow-up questionnaire were included in this study. Interview responses given by the child’s biological father, grandmother, adoptive mother, or others were excluded from the analyses. Furthermore, in the case of questionnaires that were answered twice by mothers of twins, only one set of responses was considered.

The fieldwork for the 48-month follow-up began on January 7, 2019, and ended on December 31, 2019, totaling 4,010 monitored children. Mothers answered a questionnaire about different matters, such as childcare and feeding, child health and sleep, characteristics of the mother, family and home, mother’s health and contraception, and others. The interviewers who applied these questionnaires to the mothers were trained periodically.

### Dependent Variable

The studied dependent variable referred to the contraindicated use of CHC, POC, and IUD by female contraceptive users. Contraindicated use of contraceptives was assessed in accordance with the WHO medical eligibility criteria^4^. In scenarios of various health conditions, these criteria classify the use of modern contraceptives as category 1 (no restrictions on the use of the method), category 2 (advantages of the method generally outweigh theoretical or proven risks), category 3 (theoretical or proven risks usually outweigh the advantages of using the method) or category 4 (unacceptable health risk if the method is used). Women in categories 3 and 4 were considered to be contraindicated for the use of contraceptive methods as they were at risk of complications.

The evaluated contraindications were based on respondents’ self-reports using the question “Do you have or did you have…?” regarding the morbidities of interest. The evaluated CHC types were combined oral pill and combined injectable contraceptive, and for POC they were progestogen-only pill, levonorgestrel and etonogestrel (implants), depot medroxyprogesterone acetate (injectable) norethisterone enanthate (injectable), whereas for IUD, they were levonorgestrel-releasing IUD (LNG-IUD) and copper-bearing IUD.

The contraindications evaluated for the use of CHC methods were the following: smoking when aged 35 years or more, liver tumor, acute hepatitis, previous deep vein thrombosis, acute venous thrombosis, stroke, heart attack or angina, controlled hypertension, decompensated hypertension, diabetes, migraine headache, migraine headache with aura, and breast cancer. For the use of POC were liver tumors, acute venous thrombosis, stroke, heart attack or angina, decompensated hypertension, diabetes, migraine headache with aura and breast cancer; whereas for the use of IUD were uterine fibroid, cervical cancer for copper-bearing IUD, and liver tumor, acute venous thrombosis, heart attack or angina, migraine headache with aura, uterine fibroid, cervical cancer and breast cancer for LNG-IUD. Thus, women who presented at least one of the contraindications studied (categories 3 and/or 4) for the use of CHC, POC or IUD and who were making use of these modern methods were considered to be contraindicated users of modern contraceptives.

### Covariates

The independent variables included were the following: age in years; education in years; family income in minimum monthly wages; body mass index (BMI, in kg/m^2^); indication of current use of the method (gynecologist, general practitioner at a primary healthcare unit, friend/relative, or other person); source from which the contraceptive method in use was obtained (purchased at a pharmacy; obtained from a healthcare center; donation from university; acquired by a health insurance plan; or another source); reason for choosing the contraceptive method (trustworthiness, efficacy, ease of use, cost/accessibility/free-of-charge, or other reason); and a feeling of security and adaptation to the method (yes or no).

The quantitative variables age in years (< 25; 25–29; 30–34; ≥ 35); education in years (0–4; 5–8; 9–11; ≥ 12); family income in minimum monthly wages (R$ 998.00) (≤ 1; 1.1–3.0; 3.1–6.0; 6.1–10; > 10), and body mass index (BMI in kg/m^2^) (< 25; 25–30; > 30) were categorized.

### Data Analysis

The analyses were performed using Stata, version 14.1 (Stata Corp., College Station, Texas, USA). A descriptive analysis on independent variables was performed. Next, the frequency of use of different contraceptives, the frequency of contraindications for the use of modern contraceptives, and the prevalence of contraindicated use of contraceptives according to the evaluated contraindications were determined with their respective 95% confidence intervals (95%CI). The prevalence of contraindicated use of modern contraceptives was then calculated both overall and according to the types of studied contraceptives (CHC, POC, and IUD), with their respective 95%CI. The chi-squared test for heterogeneity was used to compare the prevalence of contraindicated use of modern contraceptives between independent variables, and p values < 0.05 were considered significant.

### Ethical Statement

Before answering the questionnaire, all participants signed informed consent forms in which they agreed to participate in the follow-up and that guaranteed the absolute confidentiality of the informed data, thereby upholding ethical principles.

## RESULTS

Of the 4,010 interviews conducted at 48 months follow-up, 3,053 biological mothers were using modern contraception (Figure) which was considered the study sample. The mean age of the women in the final sample was 31.4 years (standard deviation: 6.6 years) (Table 1). Among the characteristics of the studied mothers, 47.1% had a family income from one to three minimum monthly wages (MW) and 65.8% had less than 12 years of schooling. Moreover, 57.4% of them had already been advised by a doctor on how to avoid getting pregnant and 45.8% had health insurance or a health plan. Most women (68.3%) had received an indication for the method they were using from a gynecologist (Table 1).

**Figure:**
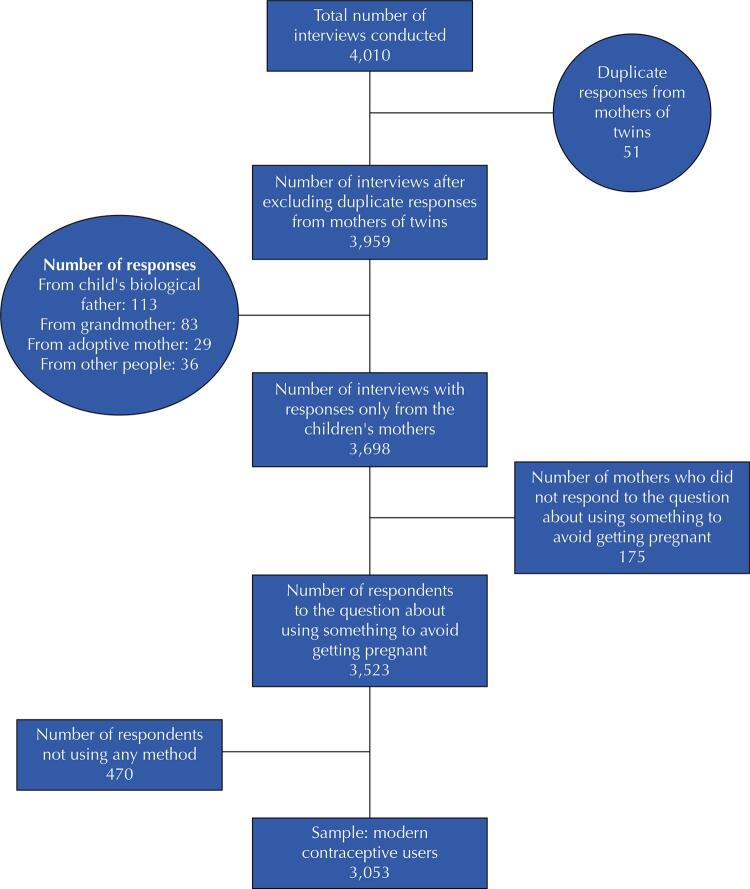
Sample selection flowchart.

**Table 1:** Sample characteristics and prevalence of contraindicated use of modern contraceptives; four-year follow-up of the 2015 Pelotas Birth Cohort.

Variables	Contraceptive users (n = 3,053)	Prevalence of contraindicated use	p-value^a^
	
	n (%)	% (95%CI)	
Age (years)			0.860
< 25	541 (17.7)	25.3 (21.8–29.2)	
25–29	737 (24.1)	25.9 (22.9–29.2)	
30–34	781 (25.6)	25.2 (22.3–28.4)	
≥ 35	994 (32.6)	26.9 (24.2–29.7)	
Family income in minimum monthly wages (R$ 998.00)			< 0.001
≤ 1	1,423 (47.1)	23.5 (19.1–28.5)	
1.1–3.0	836 (27.6)	29.2 (26.9–31.7)	
3.1–6.0	233 (7.7)	24.8 (21.9–27.8)	
6.1–10	217 (7.2)	25.3 (20.1–31.3)	
>10	315 (10.4)	14.3 (10.2–19.6)	
Education level			< 0.001
0–4	783 (25.6)	40.2 (30.8–50.4)	
5–8	1,131 (37.0)	27.8 (24.8–31.1)	
9–11	1,043 (34.2)	26.4 (23.9–29.1)	
≥12	97 (3.2)	22.6 (20.2–25.3)	
Body mass index (BMI) in kg/m^2^			0.015
< 25	1,083 (36.4)	23.2 (20.8–25.8)	
25–29	956 (32.2)	28.4 (25.6–31.3)	
≥ 30	935 (31.4)	27.6 (24.8–30.6)	
Indication for the method currently in use			< 0.001
Gynecologist	2,080 (68.3)	29.8 (27.9–31.8)	
General practitioner at PHU	230 (7.5)	26.5 (21.2–32.6)	
Friend/relative	102 (3.4)	30.4 (22.1–40.1)	
Other	634 (20.8)	12.6 (10.2–15.4)	
Source for contraceptive method in use			< 0.001
Purchase at a pharmacy	1,928 (63.6)	31.7 (29.6–33.8)	
Health center/donation from university	761 (25.1)	22.1 (19.3–25.2)	
Health insurance	112 (3.7)	3.6 (1.3–9.3)	
Other	230 (7.6)	2.2 (0.9–5.1)	
Reason for choosing the contraceptive method			< 0.001
Indication of trustworthiness	808 (26.5)	31.9 (28.8–35.2)	
Efficacy	719 (23.6)	25.2 (22.1–28.5)	
Ease of use	623 (20.4)	27.8 (24.4–31.4)	
Cost/accessibility/free-of-charge	111 (3.6)	23.4 (16.4–32.3)	
Other	790 (25.9)	19.5 (16.9–22.4)	
Feeling of security and adaptation to the method			0.085
No	355 (11.7)	29.9 (25.3–34.9)	
Yes	2,683 (88.3)	25.5 (23.9–27.2)	

PHU: primary healthcare unit; 95%CI: 95% confidence interval.

^a^Chi-squared test of heterogeneity.

Regarding the prevalence of contraindicated use of the studied modern contraceptives according to independent variables, Table 1 shows that women with a family income of between one and three MW reported the greatest prevalence (29.2%) of contraindicated use of modern contraceptives (p < 0.001). The higher the woman’s level of education, the lower the prevalence of contraindicated use of modern contraceptives, in which women with 12 years of education or more showed the lowest prevalence (22.6%; p = 0.001). Those who received the indication for the currently used method from friends/relatives and gynecologists showed greater prevalence of contraindicated use of modern contraceptives [30.4% and 29.8% respectively] (p < 0.001). Lastly, women who obtained their contraceptive method by purchasing it at a pharmacy reported the greatest prevalence of contraindicated use of modern contraceptives (31.7%; p < 0.001).

The most used contraceptive belonged to the CHC group, accounting for 41.8%, followed by 31.2% of POC. The third most used group was condoms (male and female), with 13.5% (Table 2). The proportion of women with at least one contraindication for the use of modern contraceptives was 54.8% (Table 3). Migraine headache was the most prevalent contraindication, accounting for 44.2% of them, followed by controlled hypertension (10.1%). The contraindication with the highest prevalence was migraine headache (17.9%), followed by migraine headache with aura (6.4%) and controlled hypertension (4.0%). The prevalence of contraindicated use of modern contraceptives was 25.9%.

**Table 2:** Frequency of use of different contraceptives (n = 3,053).

Contraceptive method	n	%	95%CI
Combined hormonal contraceptive	1,275	41.8	40.0–43.5
Progesterone-only contraceptive	950	31.1	29.5–32.8
Male and female condoms	411	13.5	12.3–14.7
Ligature/ligation	180	5.9	5.1–6.8
Intrauterine devices			
Copper-bearing	153	5.0	4.3–5.8
Levonorgestrel-releasing	18	0.6	0.4–0.9
Vasectomy undergone by partner	26	0.85	0.6–1.2
Rhythm (calendar)	11	0.4	0.2–0.6
Interrupted coitus	6	0.2	0.1–0.4
Emergency contraceptive pills	1	0.03	0.004–0.23
Other methods^a^	22	0.72	0.5–1.1

95%CI: 95% confidence interval.

^a^Other methods: other contraceptives that women failed to specify.

**Table 3:** Prevalence of contraindication and of contraindicated use of modern contraceptives according to contraindications; four-year follow-up of the 2015 Pelotas Birth Cohort (n = 3,053).

Variable	Prevalence of contraindications			Prevalence of the incorrect use in the presence of such contraindication		
	
	n	%	95%CI	n	%	95%CI
Contraindications						
Smoking aged ≥ 35 years	170	5.6	4.8–6.5	59	1.9	1.5–2.5
Liver tumor	6	0.2	0.09–0.4	4	0.1	0.05–0.3
Acute hepatitis	43	1.4	1.0–1.9	17	0.6	0.3–0.9
Previous deep vein thrombosis	10	0.3	0.2–0.6	4	0.1	0.05–0.3
Acute venous thrombosis	4	0.1	0.05–0.3	0	0.0	-
Stroke	3	0.1	0.03-0.3	0	0.0	-
Heart attack or angina	17	0.6	0.3–0.9	14	0.5	0.3–0.8
Controlled hypertension	306	10.1	9.0–11.2	123	4.0	3.4–4.8
Decompensated hypertension	91	3.0	2.4–3.6	5	1.8	1.4–2.3
Diabetes	69	2.3	1.8–2.9	31	1.0	0.7–1.4
Migraine headache	1,348	44.2	42.4–46.0	547	17.9	16.6–19.3
Migraine headache with aura	283	9.3	8.3–10.4	195	6.4	5.6–7.3
Uterine fibroid	87	2.9	2.3–3.5	2	0.1	0.02–0.3
Cervical cancer	2	0.1	0.02–0.3	1	0.03	0.005–0.2
Breast cancer	8	0.3	0.1–0.5	5	0.2	0.07–0.4
Number of contraindications						
1 contraindication	1,08	35.4	33.7–37.1	582	19.1	17.7–20.5
2 contraindications	451	14.8	13.6–16.1	170	5.6	4.8–6.4
3 contraindications	99	3.2	2.7–3.9	27	0.9	0.6–1.3
4 contraindications	38	1.2	0.9–1.7	12	0.4	0.2–0.7
5 contraindications	3	0.1	0.03–0.3	1	0.03	0.005–0.2
7 contraindications	1	0.03	0.005–0.2	-	-	-
Total	1,672	54.7	53.0–56.5	792	25.9	24.4–27.5

95%CI: 95% confidence interval.

Table 4 describes the prevalence of contraindicated use of each type of contraceptive according to the studied contraindications. The group of CHC had the highest prevalence of contraindicated use (52.1%), followed by progesterone-only contraceptives (13.1%) and intrauterine devices (2.3%). CHC users’ prevalence of contraindications exceeded that of users in general. For migraine headaches, the prevalence was 17.9% overall but 42.9% among CHC users. For controlled hypertension, its prevalence was 4.0% in general but 9.6% in this group.

**Table 4:** Prevalence of contraindicated use of each type of modern contraceptives according to contraindications; four-year follow-up of the 2015 Pelotas Birth Cohort (n = 3,053).

Variable	CHC among contraceptive users in general	POC among contraceptive users in general	IUDs among contraceptive users in general
		
	% (95%CI)	% (95%CI)	% (95%CI)
Evaluated contraindications (n = 3,053)			
Smoking aged ≥ 35 years	1.9 (1.5–2.5)	*	*
Liver tumor	0.1 (0.02–0.3)	0.1 (0.02–0.3)	0.0
Acute hepatitis	0.6 (0.3–0.9)	*	*
Previous deep vein thrombosis	0.1 (0.05–0.3)	*	*
Acute venous thrombosis	0.0	0.0	0.0
Stroke	0.0	0.0	*
Heart attack or angina	0.1 (0.03–0.3)	0.4 (0.2–0.7)	0.0
Controlled hypertension	4.0 (3.4–4.8)	*	*
Decompensated hypertension	1.1 (0.8–1.6)	0.6 (0.4–1.0)	*
Diabetes	0.9 (0.6–1.3)	0.2 (0.1–0.4)	*
Migraine headache	17.9 (16.6–19.3)	*	*
Migraine headache with aura	3.2 (2.6–3.9)	3.1 (2.6–3.8)	0.1 (0.02–0.3)
Uterine fibroid	*	*	0.1 (0.02–0.3)
Cervical cancer	*	*	0.03 (0.01–0.2)
Breast cancer	0.1 (0.02–0.3)	0.1 (0.03–0.3)	0.0
Prevalence of contraindicated use	21.7 (20.3–23.2)	4.1 (3.4–4.8)	0.1 (0.05–0.3)
Prevalence of contraindicated use among CHC users (n = 1,275)	52.1 (49.3–54.8)	NA	NA
Prevalence of contraindicated use among POC users (n = 950)	NA	13.1 (11.1–15.4)	NA
Prevalence of contraindicated use among IUD users (n= 171)	NA	NA	2.3 (0.9–6.1)

CHC: combined hormonal contraceptive; POC: progesterone-only contraceptives; IUD: intrauterine devices; 95%CI: 95% confidence interval; NA: not applicable.

* Failed to represent a contraindication for the contraceptive in question.

## DISCUSSION

This research described the use of different types of modern contraceptives in women who had contraindications for their use according to the WHO medical eligibility criteria and the factors associated with their inadequate use. The most widely used modern contraceptive was the combined hormonal contraceptive class, followed by progesterone-only contraceptives. In total, one in four women has used contraindicated contraceptives, and approximately one in two women with at least one contraindication used a contraindicated modern contraceptive method. Family income between 1 and 3 MW, lower education, BMI greater than 25 kg/m^2^, indication of the method in use from a friend/relative and obtaining the method in use at a healthcare center or by purchase at a pharmacy were risk factors showed greater prevalence of contraindicated use of modern contraceptive methods.

In the United States, 80% of women have used CHC at least once^20^. This corroborates the findings from this study, in which CHC was the method most used among contraceptive users. Rodríguez-Rodríguez et al.^21^ reported similar results, such that CHC was the most used, followed by POC, and with condoms in third place. However, in an investigation by Trindade et al.^22^, carried out with National Health Survey (*Pesquisa Nacional de Saúde* – PNS) data in women aged between 18 and 49 years, oral contraceptives were reported to be the most used method, accounting for 34.2%, followed by surgeries (25.9%), condoms (14.5%), and IUD last (1.8%), thus evincing no growth in condom use and a high prevalence of surgical practice. These findings can be explained by the fact that the population of this study was composed of younger women, with less possibility of opting for tubal ligation. Evidence shows that women aged between 35–49 years presenting the highest prevalence of surgical practice^22^.

The proportion of women with at least one contraindication for the use of modern contraceptives was much higher than what was reported in other works^11,12^. This can be explained by the fact that those studies only evaluated contraindications for the use of CHC, ignoring the other modern contraceptives evaluated in this work, such as POC and IUD. Moreover, those studies investigated smaller selections of contraindications for the use of CHC from among the WHO medical eligibility criteria were investigated, compared with the present investigation which evaluated several contraindication conditions to CHC. CHC is the method with more contraindications than POC and IUD^7^, which might explain this higher prevalence of contraindicated use.

This study found a high prevalence of contraindicated use of modern contraceptive methods than other studies^8,10,12,23^. However, Assiri et al.^9^ from Saudi Arabia found a prevalence of contraindicated use of combined oral contraceptives of 31.3%, slightly higher than in this investigation. Correa et al.^11^ used data from a 2008 telephone landline survey of a population sample of women aged from 18 to 49 years who were living in the capitals of the 26 Brazilian states and in the Federal District and found that the prevalence of contraindicated use of oral contraceptives among these women was 12%. Furthermore, studies carried out in 1992 and 1999, using representative population-based samples of women aged between 20 and 49 years who were living in Pelotas found that, among users of contraceptive methods, 12.4% and 12.5% use contraindicated oral contraceptives, respectively^24,25^. These prevalences may have been due to the fact that those studies only evaluated a few contraindications (hypertension, smokers aged 35 years or over, diabetes mellitus and cardiovascular disease). It is also important to note that only the use of oral contraceptives was assessed. If more contraindications had been evaluated, the prevalence of use of modern contraceptives by women who presented contraindications for their use would probably have been higher.

Migraine headache without aura was the most prevalent contraindication, thus corroborating the findings of Doshi et al.^26^ However, other studies have reported hypertension to be the most prevalent problem^11,12,27^. Some of this research evaluated women aged 18–49 years and others, women aged 20–49 years. The mean age (34 years) was greater than that of this study. Moreover, more than 70% of the sample in one of these studies^27^ was composed of overweight and obese women: this condition is a risk factor for high blood pressure. This characteristic of the sample could explain why hypertension was the most prevalent contraindication in that study. With increasing body mass index in young women in developing countries^28^, hypertension may increase in young mothers evaluated with obesity or overweight.

Among the prevalence of contraindicated use of modern contraceptives according to the independent variables, higher-income women showed the lowest prevalence of contraindicated use. Hugon-Rodin et al.^23^ also found that low-income women were more likely to be incorrectly prescribed contraceptive methods. Moreover, women with low economic status could face difficulties accessing healthcare services to seek recommendations for appropriate methods. Moura et al.^16^ (2007) evaluated the nature of family planning services in Brazil and the existence of barriers to services and found a lack of appropriate services and formal protocol, which might corroborate our results.

A way to acquire or start using a contraceptive method consists of obtaining it from a pharmacy without a mandatory prescription. Access in pharmacies is a political conquest to guarantee access but lack of information may induce errors when acquiring the method. For instance, a study by Machado et al.^29^ reported that 66% of the evaluated contraceptive users were interested in receiving more information on all methods. Additionally, self-medication may result from individual social and political processes, rather than necessarily from access to information about the method. It has been suggested that women with chronic medical conditions might be less likely to receive contraceptive counseling^30^. Another possibility for acquiring or starting to use contraceptive methods would be by consultations with healthcare providers in public or private healthcare services. However, in this study, women with a prescription for contraceptives from a gynecologist or general practitioner unexpectedly showed a greater prevalence of contraindicated use of modern methods. It had been expected that contraindicated use prevalence would be lower among these women, such that prescription or indication of contraceptive methods by healthcare providers would have acted as a protective factor against contraindicated use. A study reported that the reason gynecologists prescribed combined oral contraceptives with other methods with several contraindications included endometriosis and the perceived convenience of menstrual suppression^31^. Moreover, 93.0% of the studied gynecologists stated that patients requested the prescription of combined oral contraception^31^. This choice must be based on a health evaluation by the physician before a prescription.

The strengths of this study include that, to our knowledge, it is one of the first studies to assess the contraindicated use of modern contraception in a developing country and to estimate the prevalence of use of three types of modern contraceptives (CHC, POC, and IUD). However, this study has some limitations, one of which refers to the care that the prevalence it found not be generalized for the entire population as the sample assessed is unrepresentative of Brazil, thus external validity may not be guaranteed.

Moreover, the evaluation of contraindications was based on self-reports, rather than on medical evaluations. Thus, the prevalence of the studied contraindications may have been underestimated. Migraine without aura beginning in women less than 35 is not a contraindication (category 2) for CHC, but it is a contraindication (category 3) in women with continuous migraine without aura. Thus, this investigation had no information about the beginning of the migraine to separate each category, and all women with migraine without aura were considered contraindicated. Therefore, the prevalence of contraindicated use may have been overestimated. It is also the case for the use of CHC and POC methods in the presence of diabetes, which is category 2 for WHO if there is no nephropathy, neuropathy, or retinopathy. However, due to the low prevalence of women with diabetes as a contraindication, we believe that lack of information about this disease should not interfere significantly with the prevalence of modern contraceptive use. Another important limitation was the inability to identify who would be in the “other” categories of the variables “indication for the method in use”, “source from which the method was obtained,” and “reason for choice” since individuals in these categories showed references with a lesser prevalence of contraindicated use of modern contraceptives. Further, one great limitation is the studied population. Since only mothers were included, nulliparous women were excluded. This group could be younger and has been shown to have less access to health care services and use contraceptive methods less and more inappropriately than parous ones.

## CONCLUSION

In total, one in four women using contraceptives did so despite showing at least one contraindication for the method in use. Access to primary healthcare for mothers of young children is greatly facilitated in Pelotas, the city in which this study was carried out, given the large dimensions of the public and private healthcare service provision in this city. The findings, therefore, indicate an important flaw in the offered healthcare process. It is important to plan a policy with a focus on training healthcare providers on the prescription and appropriate indication of contraception for women with different contraindications to their use, as well as policies aimed at the general population to explain appropriate modern contraceptive use.
